# The Effect of Femtosecond Laser Irradiation and Plasmon Field on the Degree of Conversion of a UDMA-TEGDMA Copolymer Nanocomposite Doped with Gold Nanorods

**DOI:** 10.3390/ijms232113575

**Published:** 2022-11-05

**Authors:** Attila Bonyár, Melinda Szalóki, Alexandra Borók, István Rigó, Judit Kámán, Shereen Zangana, Miklós Veres, Péter Rácz, Márk Aladi, Miklós Ákos Kedves, Ágnes Szokol, Péter Petrik, Zsolt Fogarassy, Kolos Molnár, Mária Csete, András Szenes, Emese Tóth, Dávid Vas, István Papp, Gábor Galbács, László P. Csernai, Tamás S. Biró, Norbert Kroó, NAPLIFE Collaboration

**Affiliations:** 1 Department of Electronics Technology, Faculty of Electrical Engineering and Informatics, Budapest University for Economics and Informatics, 1111 Budapest, Hungary; 2 Department of Biomaterials and Prosthetic Dentistry, Faculty of Dentistry, University of Debrecen, 4032 Debrecen, Hungary; 3 Wigner Research Centre for Physics, 1525 Budapest, Hungary; 4 Centre for Energy Research, Institute of Technical Physics and Materials Science (MFA), 1121 Budapest, Hungary; 5 Department of Polymer Engineering, Faculty of Mechanical Engineering, Budapest University of Technology and Economics, 1111 Budapest, Hungary; 6 Department of Optics and Quantum Electronics, University of Szeged, 6720 Szeged, Hungary; 7 Department of Inorganic and Analytical Chemistry, University of Szeged, 6720 Szeged, Hungary; 8 Department of Physics and Technology, University of Bergen, 5007 Bergen, Norway; 9 Frankfurt Institute for Advanced Studies, 60438 Frankfurt/Main, Germany; 10 Hungarian Academy of Sciences, 1051 Budapest, Hungary

**Keywords:** nanocomposite, UDMA, plasmonics, femtosecond laser, nanorod

## Abstract

In this work, the effects of femtosecond laser irradiation and doping with plasmonic gold nanorods on the degree of conversion (*DC*) of a urethane dimethacrylate (UDMA)–triethylene glycol dimethacrylate (TEGDMA) nanocomposite were investigated. The UDMA-TEGDMA photopolymer was prepared in a 3:1 weight ratio and doped with dodecanethiol- (DDT) capped gold nanorods of 25 × 75 or 25 × 85 nm nominal diameter and length. It was found that the presence of the gold nanorods alone (without direct plasmonic excitation) can increase the *DC* of the photopolymer by 6–15%. This increase was found to be similar to what could be achieved with a control heat treatment of 30 min at 180 °C. It was also shown that femtosecond laser impulses (795 nm, 5 mJ pulse energy, 50 fs pulse length, 2.83 Jcm^−2^ fluence), applied after the photopolymerization under a standard dental curing lamp, can cause a 2–7% increase in the *DC* of undoped samples, even after thermal pre-treatment. The best *DC* values (12–15% increase) were obtained with combined nanorod doping and subsequent laser irradiation close to the plasmon resonance peak of the nanorods (760–800 nm), which proves that the excited plasmon field can directly facilitate double bond breakage (without thermoplasmonic effects due to the short pulse length) and increase the crosslink density independently from the initial photopolymerization process.

## 1. Introduction

Urethane dimethacrylates (UDMA) is one of the most widely used monomers in dental composites along with low-molecular-weight triethylene-glycol dimethacrylate (TEGDMA) and ethylene glycol derivative diluent. The continuous improvement of such resin-based dental materials usually focuses on a matrix with low polymerization shrinkage and a better degree of conversion—especially in the deeper regions of the applied filler—along with improved mechanical properties and biocompatibility [1,2].

The most often used parameter to quantify the network structure of dimethacrylate polymers is the degree of double-bond conversion (*DC*). *DC* is used to characterize the extent of polymerization by comparing the amount of remaining methacrylate double bonds (C=C) to the initial amount. The *DC* in poly(dimethacrylate)s is never 100%; most composite dental materials usually reach a *DC* of around 50–75% [3,4,5]. A *DC* below 50% is generally considered unacceptable for most practical applications [6], but for dentistry, some authors suggest a minimum of 55% [7] to avoid the presence of a sol fraction in the polymer. *DC* can be measured by several analytical methods, such as Fourier Transform Infrared (FTIR) Spectroscopy [8,9], Raman Spectroscopy (RS) [5,10], Differential Scanning Calorimetry (DSC) [11] or Solid State Nuclear Magnetic Resonance (ssNMR) [12].

Increasing the *DC* of a methacrylate polymer improves many of its macroscopic properties, such as hardness [13,14], biocompatibility [8,15], a tendency to yellowing, etc. However, maximizing the *DC* is not a trivial task, as it depends on many material parameters and experimental conditions (such as monomer composition, temperature, irradiation intensity and time, initiator concentration, co-initiator/inhibitor system etc.). Noble metal nanoparticles made of gold and silver were successfully used in the past to increase the *DC* of methacrylate and epoxy resins upon polymerization [16]. Their effect was usually attributed to the plasmonic heating of nanoparticles embedded into the polymer matrix during photopolymerization [17].

The effects that occur during the interaction of femtosecond laser pulses with gold nanoparticles have been previously studied with both experimental and theoretical methods [18,19,20,21]. This interaction involves energy and energy dissipation and could eventually result in the melting of the nanoparticle. It was found that when the metallic nanoparticle is exposed to an ultrafast laser pulse, the energy of photons is absorbed by the free electrons. The electronic gas thermalizes rapidly to a Fermi-Dirac distribution over a 50–100 fs time scale [18]. This is followed by the increase of the lattice temperature due to electron-phonon scattering in ~1.7 ps [18], resulting in a thermal equilibrium between the electrons and the lattice. With the increase of the particle temperature, energy exchange starts between the lattice and the surrounding medium through phonon-phonon coupling, leading to a thermal equilibrium within 0.1–1 ns [21].

According to the theoretical studies, in the case of single-shot irradiation, the temperature of the nanoparticle could reach above 1000 K, resulting in a temperature of a few hundred Kelvin of the medium (water) at the particle surface [21]. However, this temperature drops rapidly in 50–100 ps, and the thermal penetration depth for a 48 × 14 nm particle was found to be 20–25 nm from the nanoparticle surface [21]. These findings indicate that, in the case of a single ultrashort femtosecond laser shot, the thermal effect is limited to the vicinity of the nanoparticle.

The fabricated samples were also used as target materials for the purpose of the NanoPlasmonic Laser Induced Fusion Energy (NAPLIFE) project [22]. The ultimate aim of the project is to apply seven thin layers of 21 μm thickness on the target, with a single laser pulse just crossing the target once [22]. This is to avoid thermalization and keep non-thermal, beam-directed dynamics without isotropization. In this case, the absorbed energy and momentum in the nanorod antennas will take the form of resonant plasmon-polaritons, and this energy and momentum are carried by the electrons. Due to the strong field, the electrons will leave the nanorod antennas [23]. This relatively short lifetime of about 100 fs is sufficient for the initial ignition of the target and the acceleration of the protons by the ejected electrons, but not for the ohmic heating of the antennas (needing ps time scales). At the energies of the ignition laser pulse, we do not expect the nanorod antennas to stay intact for picoseconds. Our dynamics are fundamentally non-thermal following ref. [24] or the Double-Cone ignition [25], with moderate thermalization.

This work aimed to investigate the effects of the irradiation with a single femtosecond laser pulse and the presence of gold nanorods resonant with the laser wavelength upon the degree of conversion of a UDMA-TEGDMA nanocomposite (weight ratio of 3:1). For comparative purposes, the laser treatment experiments are also executed on samples that are subjected to thermal treatment (conventional heating). The main novelty of our work with respect to previous studies in the field is the following:

(1) In previous works, the effects of nanoparticle doping and plasmonic resonance were investigated while the nanoparticles were excited during the photopolymerization process [13,15,17,26]. This was carried out by using simultaneous excitation upon photopolymerization, either with the same excitation source or by using separate sources, e.g., blue light to activate the photoinitiator (camphorquinone, CQ) and green to excite gold nanoparticles [17]. In our case, the resonant wavelength of the nanorods is in the red–infrared region (plasmonic absorption peak between 760 and 800 nm, as will be shown later). Since only a standard dental curing lamp that emits blue light was used for photopolymerization [27], the plasmon resonance on the nanorods was not directly excited during the photopolymerization process.

(2) The nanorods were excited with a single femtosecond laser pulse (795 nm) close to their resonant wavelength after the photopolymerization. The plasmonic effects of the nanorods are thus independently investigated from the photopolymerization process. It has to be emphasized that, due to the single shot, the short pulse and the low concentration of the gold nanoparticles mean thermalization will be limited to tiny regions around the nanorods; this way, the effect of the strong plasmon field of the breakage of methacrylate bonds and crosslinking formation can be distinguished from thermoplasmonic effects.

(3) The effect of a high-energy femtosecond laser pulse on the UDMA-TEGDMA copolymer was independently studied as well (without the presence of nanorods).

## 2. Results

### 2.1. Nanocomposite Characterization

**Figure 1:**
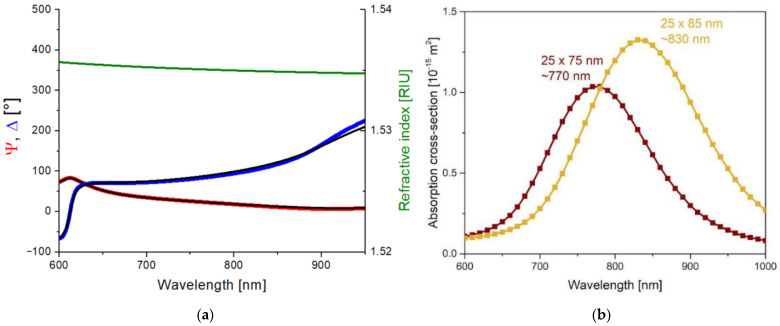
(**a**) Scanning ellipsometry results measured on a thin (~380 nm) UDMA-TEGDMA layer without nanorods and prepared on a silicon wafer. The blue and red curves are the measured (Ψ, Δ) values, and the black curves are the fitted optical model. The green curve represents the Cauchy dispersion of the obtained refractive index in the relevant wavelength range. (**b**) Calculated absorption spectra cross-section of the 25 × 75 and 25 × 85 nanorods in the UDMA matrix (COMSOL simulation results).

In order to investigate the effect of the plasmon field on the polymer structure upon laser irradiation, the absorption of the gold nanorods had to be tuned to match the excitation, that is, the emission wavelength of the laser at 795 nm. Since the absorption properties of the nanorods depend on the dielectric properties of the surrounding medium, the refractive index of the UDMA-TEGDMA matrix has to be known to estimate the optimal nanorod size for our excitation. For this purpose, the complex refractive index of the polymer matrix was determined by using scanning ellipsometry on the bare UDMA-TEGDMA mixture after photopolymerization. A thin layer of polymer (~380 nm) was prepared on a silicon wafer for these measurements. Figure 1a presents the obtained Ψ and Δ values. A three-parameter Cauchy dispersion model was fitted on the measured data, as per Equation (1). The real part of the resulting refractive index (*n*) is also given in Figure 1a. In the relevant wavelength range, the refractive index of the matrix is around 1.535.

(1) $$n=A+\frac{B}{\lambda^{2}}+\frac{C}{\lambda^{4}}$$

This value was used to model the dielectric medium around the nanorods in COMSOL Multiphysics. Based on the simulations, the −emission wavelength of the laser (795 nm) falls between the absorbance peaks of 25 × 75 nm and 25 × 85 nm nanorods, as can be seen in Figure 1b. For this reason, nanorods of both sizes were used to create nanocomposites. It has to be mentioned that, in addition to the nominal values of the nanorods, the size distribution variations (±10% for both axes, according to the manufacturer) were also taken into consideration.

**Figure 2:**
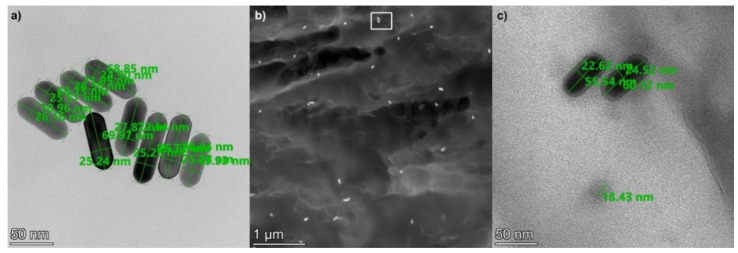
(**a**) STEM image of the 25 × 75 gold nanorods (on a carbon filter). (**b**) HAADF STEM image of the implanted nanorods inside the UDMA-TEGDMA polymer matrix. (**c**) HRTEM image of nanoparticles inside the polymer matrix, the area corresponds with the white rectangle (rotated by 90 degrees) in (**b**). The bottom circular shape with an 18.5 nm diameter corresponds to an Sn nanoparticle.

In order to check the homogeneity of their implantation into the polymer matrix, scanning transmission electron microscopy (STEM) was used. Figure 2 presents STEM images of the 25 × 75 gold nanorods after implantation into the UDMA-TEGDMA polymer matrix at a 1.9 × 10^12^ mL^−1^ concentration. The size of the nanorods was measured on images where the nanorods were fixed to a carbon filter, such as in Figure 2a. Based on 25 measured rods, the average (and standard deviation) of their longer and shorter axes are 76 (±8) and 26 (±2) nm, respectively. To measure the size of the nanorods, only those who had both ends in focus were considered. This 8–10.5% deviation from the nominal values is within the specification of the manufacturer and complies with the expected characteristics of the fabrication technology.

**Figure 3:**
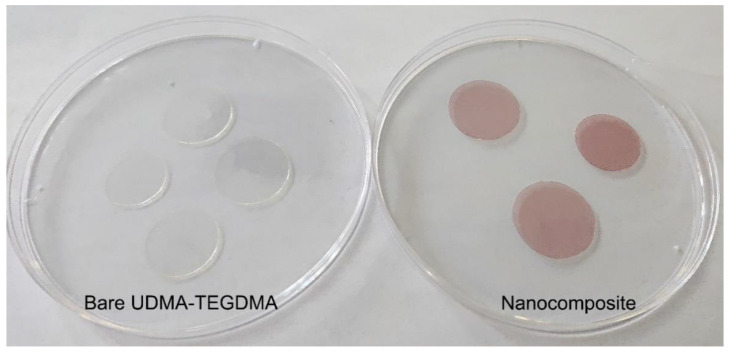
Photographs of bare (left) and Au nanorod-doped (right) photopolymerized resin samples, created with the preparation steps illustrated in Section 4.1. In this photo, the doped resin samples contain 25 × 75 nm nanorods at a 1.9 × 10^12^ mL^−1^ concentration.

The distribution of the nanorods is investigated based on high-angle annular dark-field (HAADF) STEM images, such as the one shown in Figure 2b. The density of the gold nanorods inside the polymer matrix was estimated to be between 9 and 20 μm^−3^. It can be seen in Figure 2b that the nanorods are evenly distributed inside the matrix without any sign of extensive particle aggregation. This corresponds well with the optical observations (see Figure 3). Note that inside the polymer matrix, the orientation of the rods is random; thus, their full length cannot always be observed on a cross-sectional STEM image. It is interesting to add that many small spherical Sn nanoparticles were also observed in the nanocomposite. As seen in Figure 2c, the diameter of these particles is between 18 and 20 nm, and their density inside the matrix is estimated between 60 and 130 μm^−3^. These nanoparticles originate from the UDMA-TEGDMA matrix, where Sn is used as a catalyst during its synthesis [28].

**Figure 4:**
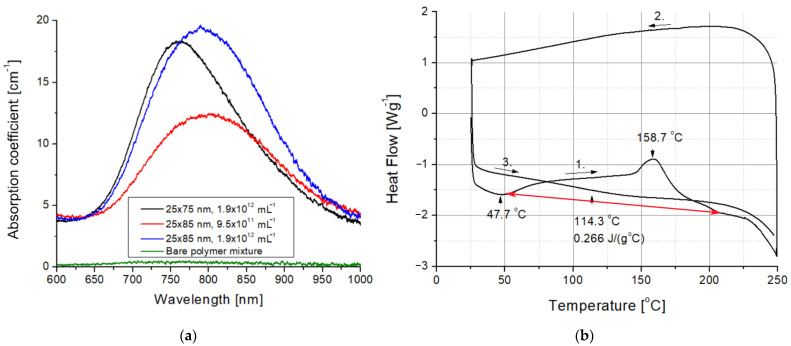
(**a**) Optical spectra of the bare UDMA-TEGDMA polymer mixture and the different nanocomposites. (**b**) Differential scanning calorimetry results measured on a bare UDMA-TEGDMA resin sample without nanorods. The red arrow marks the baseline for peak integration, whereas the black arrows with numbers indicate the direction and the serial number of thermal half cycles.

The presence of the gold nanorods in the nanocomposites is visible to even the naked eye. As can be seen in Figure 3, while the bare UDMA-TEGDMA polymer is nearly completely transparent in the visible range, the nanocomposite samples have a distinct crimson (dark pink) color. Based on the measured absorbance spectra presented in Figure 4a, the LSPR peak of the 25 × 75 nanorods in the polymer matrix is around 760–765 nm, while the peak of 25 × 85 nanorods is around 795–800 nm. The absorption peak positions are located at slightly lower wavelengths than what was estimated by the numerical simulation (Figure 1b). Since the variation of size distributions was also taken into consideration (±10% for both axes according to the manufacturer), the FWHM of the spectra is only slightly wider than anticipated. However, most importantly, it can be stated that both types of nanorods are active in the polymer matrix at the emission wavelength of the laser (795 nm). The absorption coefficient (*α*) of the nanocomposite at this wavelength was calculated by using the known value of sample thickness (164 μm). Based on this, at the emission wavelength, the *α* of the nanocomposite with 25 × 75 nanorods (1.9 × 10^12^ mL^−1^) is around 16.5 cm^−1^, while it is 12.3 and 19.2 cm^−1^ for the 25 × 85 nanocomposites at the two tested concentrations (9.5 × 10^11^ mL^−1^ and 1.9 × 10^12^ mL^−1^, respectively). As a comparison, the bare polymer matrix has an *α* of 0.4 cm^−1^ at this wavelength.

In order to explain the effects of femtosecond laser irradiation on the structure of the nanocomposite, DSC measurements were also performed on the bare UDMA-TEGDMA resin after photopolymerization. The results of a heating-cooling-heating cycle between 15 and 250 °C with ramps of 10 °C·min^−1^ are presented in Figure 4b. In the first heating cycle, two characteristic peaks can be observed. The first peak at 47.7 °C indicates an endotherm process, which is the evaporation of the absorbed water from the polymer matrix. The second peak at 158.7 °C marks the breaking of C=C double bonds and the formation of crosslinks between polymer chains (additional polymerization), which is an exotherm process. The degradation of the polymer matrix starts at around 230 °C. The glass transition temperature (*T*_g_) and the specific heat capacity (*c_p_*) of the polymer were established based on the third cycle as *T*_g_ = 114.3 °C and *c_p_* = 0.266 J·g^−1^·°C^−1^.

By integrating the exotherm peak in Figure 4b, the degree of conversion for the initial polymer after photopolymerization can be calculated based on Equations (2) and (3) [3,29,30].

(2) $$DC\left(\%\right)=\left(1-\frac{\Delta H_{p}}{\Delta H_{100}}\right)\times 100$$

(3) $$\Delta H_{p}=\frac{\Delta H_{p,exp}\times MW}{f}$$

*MW* is the molecular weight of the monomer (*MW* = 424.08 g·mol^−1^, UDMA = 470 g·mol^−1^, TEGDMA = 286.32 g·mol^−1^, and weight ratio = 3:1), *f* is the number of functional groups in the monomers (*f* = 2 in both dimethacrylates used here). $\Delta H_{p}$ is the molar enthalpy determined from the DSC experiments ($\Delta H_{p,exp}$) as in Equation (2), while $\Delta H_{100}\;$ is the molar enthalpy of polymerization for the theoretical case of total conversion (57.8 kJ·mol^−1^ [31]). Based on these, the *DC* of the UDMA-TEGDMA nanocomposite is estimated to be from 71–72% after photopolymerization. This value is higher compared with our previous experience with this polymer (55–62%, [14]); however, a thin layer was used for polymerization, not a bulk material. During the second heating ramp shown in Figure 4b, no exotherm peak could be observed, and the heat of the first cycle was enough to stabilize the conversion of the polymer matrix.

**Figure 5:**
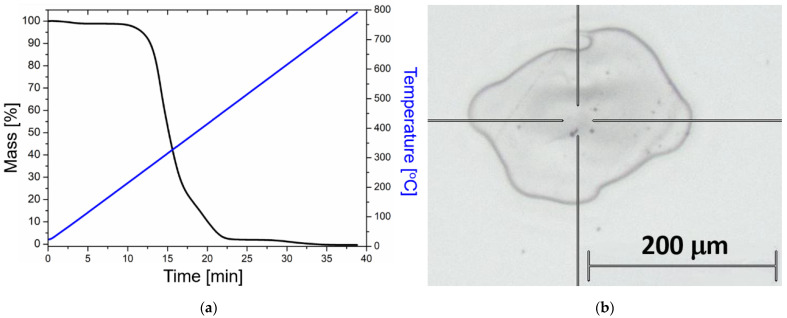
(**a**) Thermogravimetric analysis (TGA) performed on the UDMA-TEGDMA resin after photopolymerization. Blue: temperature ramp. Black: TGA curve. (**b**) Optical microscopy image of a typical crater after laser irradiation (undoped sample), where the Raman measurements were performed at the edges of the crater.

To further examine the thermal stability of the material, TGA was performed on the polymerized mixture. As can be seen in Figure 5a, the degradation starts at around 230 °C, in good correspondence with the DSC measurements.

### 2.2. Irradiation Experiments and Raman Spectroscopy

The primary aim of the laser irradiation experiments was to investigate the effect of nanorods on the conversion of the UDMA-TEGDMA polymer. Reference samples were created where the conversion was completed by applying a heat treatment of 30 min at 180 °C to ascertain their effect on *DC*. The DSC curve of the heat-treated samples shows no exotherm peak (see Supplementary Information Figure S1).

These five investigated experimental conditions (presence of nanorods in the polymer, size and concentration of the nanorods, laser irradiation, and heat treatment) created 32 (2^5^) different cases (but for only two different concentrations). In order to reduce the parameter space, these conditions were tested in pairs, as indicated in Table 1.

**Figure 6:**
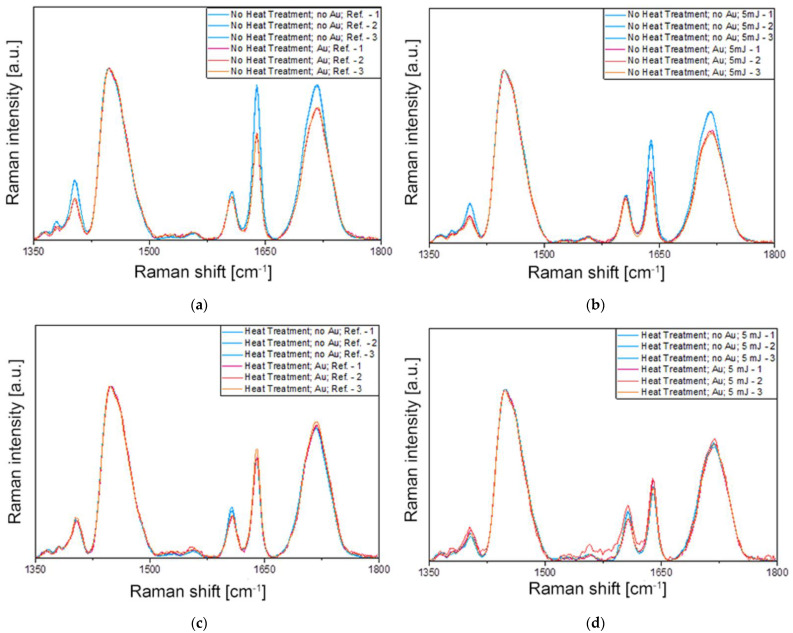
Raman spectroscopy results were measured under eight different conditions (#1–#8), which are compared in pairs in graphs (**a**–**d**) (the assignments are defined in Table 1). (**a**) Samples without heat treatment, without laser irradiation, and without and with Au NRs (#1 vs. #2). (**b**) Samples without heat treatment, with 5 mJ laser irradiation, and without and with Au NRs (#3 vs. #4). (**c**) Samples with heat treatment, without laser irradiation, and without and with Au NRs (#5 vs. #6). (**d**) Samples with heat treatment, with 5 mJ laser irradiation, and without and with Au NRs (#7 vs. #8). In every panel, three spectra measured on different spots on the surface are given for each condition.

The effect of heat treatment was tested with the 25 × 75 nanorods, while the effect of concentration was tested with the 25 × 85 batches. The 12 different samples were thus exposed to femtosecond laser irradiation (as discussed in Section 4.3) and subsequently subjected to Raman spectroscopy investigations. Figure 6 collects the obtained Raman results; three spectra were measured at the edges of the crater (see Figure 5b) and presented for each sample, and the experimental conditions were compared in pairs. It has to be noted that Raman measurements were also performed in the center of the craters, and they show a very small difference (<3%) compared to the edge regions.

The most important peak present in the spectra of Figure 6 corresponds to the methacrylate C=C stretching mode at 1640 cm^−1^ involved in the polymerization process. The Raman intensity of this band is used to determine the *DC* of the methacrylate-based polymers, together with another Raman peak of the monomer not affected by the polymerization reaction. In most cases, this reference peak is the aromatic C=C stretching mode of the monomer [32], except when it does not contain an aromatic ring, such as in UDMA or TEGDMA. Although a Raman peak can be observed in the spectra in Figure 6 around 1610 cm^−1^, it does not arise from the methacrylates and hence cannot be used for the calculation of the *DC*. In such cases, another Raman band of the monomer is selected as a reference, such as the C–H bond at 1447 cm^−1^, that does not suffer any chemical modification during the polymerization process [33]. Therefore, the intensity ratio of the methacrylate C=C stretching mode at 1640 cm^−1^ to the C–H peak at 1447 cm^−1^ is used for the determination of the *DC*. Lorentzian functions were fitted on the measured spectra at these two positions (see Supplementary Information Figure S2, Figure S3, Figure S4, Figure S5, Figure S6, Figure S7, Figure S8 and Table S1), and the obtained peak intensities and integrated analytical areas were used for calculating the ratios and *DC*. The average standard deviation of peak intensities measured in different spots is below 3%, considering all measurements. In the Supplementary Materials, the averaged spectra are presented for all 12 tested experimental conditions with the fitted Lorentzian functions on the evaluated peaks, including the reference polymer mixture before photopolymerization.

## 3. Discussion

Table 2 collects and compares the properties of the characteristic Raman peaks for all investigated experimental conditions. The DC for each case is calculated based on the measured intensity ratios (of the peaks at 1640 cm^−1^ and 1447 cm^−1^) compared to the reference, which was measured on the bare UDMA-TEGDMA polymer mixture before photopolymerization, as in Equation (4).

(4) $$DC=\left(1-\frac{{\left(\frac{I_{1640}}{I_{1447}}\right)}_{exp}}{{\left(\frac{I_{1640}}{I_{1447}}\right)}_{ref}}\right)\times 100\%$$

Based on the results in Table 2, the following observations can be made regarding the effect of laser irradiation, heat treatment, and the plasmon field of gold nanorods in different sizes and concentrations on the DC of the polymer composite.

***Initial DC of the photopolymer***. The initial *DC* of the UDMA-TEGDMA polymer mixture after photopolymerization (sample #1) was quite high, as it was 78.5%, which is ~7% higher compared to the estimations based on the DSC measurement. Although such high *DC*s are not unrealistic for such a thin layer exposed to high intensities during photopolymerization, and even higher *DC*s (above 95%) were obtained for other methacrylates after gamma-radiation initiated polymerization [34], this difference might originate from measurement artifacts as well. Aside from the fact that both measurement techniques are prone to variations originating from the evaluation (e.g., the integration range in the DSC measurements, Lorentzian curve-fitting on the Raman spectra), a possible cause for this difference could be the light sensitivity of the reference polymer mixture. Although maximum care was taken during the Raman measurements in order to avoid the exposure of the polymer mixture, even minimal exposure from the ambient or the laser excitation (532 nm) could initiate crosslinking and cause a drop in the intensity of the methacrylate peak (at 1640 cm^−1^), increasing the measured *DC* (Equation (4)). Another reason could be the difference between the scope of the DSC and Raman methods: the latter gives information on the *DC* in the upper 1–2 μm thick layer of the polymer, which has received the highest light intensity during the photopolymerization, in contrast, the *DC* value obtained with DSC characterizes to the whole (bulk) sample. It must be mentioned that dental lamps’ curing depth is in the several mm range [35].

***Femtosecond laser irradiation after polymerization***. The femtosecond laser pulses itself has always increased the *DC*, compared with the corresponding reference samples and regardless of heat treatment or gold nanorod doping. Without heat treatment or nanorods, the *DC* increased by 7.2% upon laser irradiation (#1 vs. #3), while without heat treatment but with nanorods, the *DC* showed a 2–5% increase, depending on the nanorod size and concentration (#2 vs. #4; #9 vs. #10; #11 vs. #12). When heat treatment was applied to the samples, there was no significant difference between the nanorod-doped and undoped samples, as both showed a similar increase (~4%) in *DC* upon irradiation (#5 vs. #7 and #6 vs. #8).

***Photopolymerization in the presence of gold nanorods.*** By comparing sample #1 with either #2, #9, or #11, we can see that the presence of the DDT-capped gold nanorods alone during photopolymerization can increase the *DC* significantly, between 6 and 15%, without any heat treatment or laser irradiation. The *DC* measured on sample #2 with the nanorods is in the same range achieved by the thermal treatment (#5 and #6) for the 25 × 75 nanorods. This result suggests that the presence of the gold nanorods alone can cause a similar amount of *DC* increase as would a subsequent heat treatment on the polymer. This result might be peculiar, especially in light of the fact that plasmon resonance was not excited directly in the gold nanorods during photopolymerization. The longitudinal plasmon absorption band of the nanorods in the polymer (see Figure 4a) is far from the blue light of the dental curing lamp used for polymerization. The transverse resonance peak of the gold nanorods, around 520–530 nm [36], is closer and could be held accountable; however, the emission spectrum of the dental curing lamp cuts abruptly at 510 nm [37], so the transverse excitation of the nanorods can also be considered negligible. The emission spectrum of the used QTH lamp is shown in Figure S9 in the Supplementary Information. On the other hand, as seen in Figure 4a, the absorbance of the polymer is increased in the blue region as well (compared to the undoped, bare matrix), and increased absorption of the nanorods can lead to a local temperature increase during the 3 min photopolymerization process [38]. The increased *DC* due to thermal processes upon photopolymerization is consistent with previous works that investigated NP-doped methacrylate systems [26].

***Excited plasmon resonance of the gold nanorods*:** Gold nanorod doping combined with plasmon excitation has a positive influence on the *DC*, compared to the corresponding references. The nanorod-doped samples always had an increased *DC* upon laser irradiation, between 2 and 5%, demonstrating the effect of the plasmon field in catalyzing the methacrylate double bond breaking and crosslinking. Plasmon resonance-initiated polymerization was reported previously, even without the presence of photoinitiators [39]. The two main proposed mechanisms are plasmon resonance energy transfer [40] and plasmon-induced charge transfer, which injects hot electrons from the gold nanoparticle into the polymer [41,42]. The hot electron can be transferred into the monomer to initiate free radicals, as proposed in [39], and can induce polymer chain growth. It has to be emphasized that the short duration of the pulse does not permit the thermalization of the system, so the observed effect is different from the thermal heating induced by the nanorods. This is also confirmed by the fact that laser irradiation on nanorod-doped samples increased the *DC* of samples that underwent thermal treatment as a control (samples #6 and #8).

***Nanoparticle size and concentration*.** Increasing either the size or the concentration of the nanorods increased the measured *DC*s, both in the irradiated and reference samples. For the reference samples (without plasmon excitation), this can be attributed to the increased surface area. After irradiation, the nanorods that had a plasmon resonance absorption band closer to the laser excitation wavelength had a higher effect on the overall *DC*. The best *DC*s obtained with 25 × 75 nanorods is 90.3% (sample #4), which is 93.7% with 25 × 85 nanorods (sample #12). This indicates that the excited gold nanorods have a higher effect compared to laser irradiation alone (#3) or nanorods without plasmonic activation (#2). The effect of the nanorods’ plasmon field on catalyzing polymerization is most pronounced by comparing samples #3 and #4 for the 25 × 75 nanorods and samples #11 and #12 for the 25 × 85 nanorods. The negative controls tested on the heat-treated samples (samples #7 and #8) also confirm this effect.

## 4. Materials and Methods

### 4.1. Materials and Sample Preparation Procedures

Dodecanethiol-capped gold nanorods (Au-DDT) in two sizes (25 nm diameter and 75 or 85 nm length) were purchased from Nanopartz Inc., Loveland, CO, USA (No. B12-25-700-1DDT-TOL-50-0.25, No. B12-25-750-1DDT-TOL-50-0.25). For the sake of simplicity, the two different types of nanorods will be referred to as 25 × 75 and 25 × 85 from now on in the text. The concentration of the nanorods was specified by the manufacturer as 12 and 10 mg·mL^−1^ in water for the 25 × 75 and 25 × 85 nanorods, respectively. We used these values to calculate the nominal nanorod concentration of the prepared nanocomposites.

The photopolymerizable dimethacrylate resin mixture consists of urethane dimethacrylate (UDMA) (Sigma Aldrich Co., St. Louis, MO, USA) and triethylene glycol dimethacrylate (TEGDMA) (Sigma Aldrich Co., St. Louis, MO, USA). The weight ratio of UDMA:TEGDMA was 3:1.

An amount of 1.5 g urethane dimethacrylate was weighed into a 100 mL round bottom glass flask, and the Au nanorods were added to the UDMA from the stock solution. Homogenization was achieved by applying sonication in an ultrasonic bath for 5 min. Next, 0.5 g TEGDMA was weighed and added to the UDMA Au nanorod mixture. The photoinitiator used was camphorquinone (CQ, 0.2 m/m%) (Sigma Aldrich Co., St. Louis, MO, USA), along with its co-initiator, ethyl-4-dimethylaminobenzoate (EDAB, 0.4 m/m%) (Sigma Aldrich Co., St. Louis, MO, USA). The CQ/EDAB ratio and concentration were maintained constant for all samples. The photoinitiator system was dissolved in the resin matrix by stirring overnight at room temperature. The nominal concentration of the 25 × 75 nanorods in the resin was 1.9 × 10^12^ mL^−1^, which equals 0.108 m/m%. As for the 25 × 85 nanorod-containing samples, two concentrations were set: 1.9 × 10^12^ mL^−1^ (0.123 m/m%) and 9.5 × 10^11^ mL^−1^ (0.062 m/m%).

**Figure 7:**
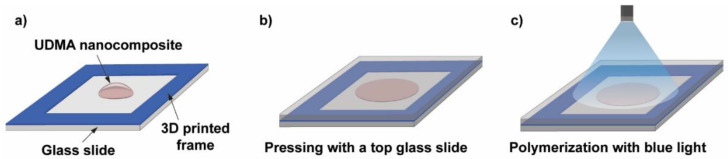
Schematic illustration of the photopolymer sample preparation steps. (**a**) A 3D-printed frame is glued onto a glass slide, which acts as a spacer, and a droplet of the UDMA-TEGDMA resin is deposited onto the glass slide. (**b**) Placing and pressing with a top glass slide onto the nanocomposite droplet. (**c**) Illumination with a blue light beam from a dental curing lamp for photopolymerization.

Figure 7 illustrates the procedure of the photopolymerization process. The essence of the process is that a small volume of the nanocomposite resin is deposited onto a glass slide equipped with a 3D-printed frame (height is 164 μm, printed by a Photon Mono X printer (Anycubic, Shenzhen, China)) prior to the deposition and another glass slide was placed on top of the construction. The frame in the construction acted like a spacer and ensured that the polymer sample discs had a constant thickness, whereas the top glass slide helped to produce an even and smooth sample surface. The thickness of the samples was later monitored by using an Alpha Step 500 surface profiler (Tencor Instruments, Milpitas, CA, USA). A standard dental curing lamp (quartz-tungsten-halogen light source (QTH) [37]) emitting blue light was used to photopolymerize the discs with a constant 3 min exposure time. The end of the fiberoptic light guide was 5 cm away from the sample during polymerization, which ensured the exposure of the whole sample, as illustrated in Figure 7.

### 4.2. Sample Characterization Methods

A Themis STEM (Thermo Scientific, Waltham, MA, USA) operated at 200 keV acceleration voltage was used to investigate the gold nanorods’ size and shape in their stock suspension, as well as their distribution within the resin. Standard Ar ion milling was used on cured polymer nanocomposite samples prior to STEM measurements.

Vis-NIR absorption spectroscopy was performed with an Avaspec 2048-4DT spectrometer (Avantes, Apeldoorn, NL). An Avantes Avalight DHS halogen light source was used for the measurements, and the spectra were recorded in the 600–1000 nm range with a resolution of 0.28 nm.

Differential scanning calorimetry (DSC) experiments were performed on a Q2000 instrument (TA Instruments, New Castle, DE, USA). A heating-cooling-heating cycle was applied between 25 and 250 °C with ramps of 10 °C/min. A 4.79 mg piece of the bare UDMA-TEGDMA matrix (without nanoparticles) was pre-polymerized, and a 4.79 mg piece was used for DSC in an Al sample holder (measured by a precision laboratory balance).

The refractive index of the UDMA-TEGDMA polymer was measured by an M-2000DI (J. A. Woollam, Lincoln, NE, USA) rotating compensator spectroscopic ellipsometer in a reflectance configuration. For this purpose, a separate, thin layer (~380 nm) of the polymer was prepared on a silicon wafer. The directly measured quantity is the complex reflection coefficient *r*_p_/*r*_s_ = tan(Ψ)exp(*i*Δ), where *r*_p_ and *r*_s_ are the reflection coefficients of light polarized parallel and perpendicular to the plane of incidence, respectively. Ψ and Δ spectra were recorded and fitted using the one-layer (silicon substrate) model.

A Renishaw (Wotton-under-Edge, UK) InVia micro-Raman spectrometer connected to a LeicaDM2700 microscope was used to characterize the bonding configuration and the chemical changes in the polymer samples upon irradiation. The equipment measures the Raman spectrum in backscattering geometry. The Raman spectra were recorded by using 532 nm excitation, and the laser was focused into a spot of ~1.3 μm diameter on the sample surface by using an objective of 50X magnification and 0.5 NA. The laser power was ~6 mW which equals 5–10% of the maximum intensity of the laser source. The accumulation time was 10 s, and spectra were recorded in the 200–3600 cm^−1^ spectral region. Before the measurements, calibration was performed by using a silicon wafer and its characteristic peak at 520 cm^−1^ Raman shift. Baseline correction and normalization of the spectra were completed in the Renishaw WIRE software, while the fitting of the Raman peaks with a Lorentzian function was performed in the Origin 2019 (Originlab, Northampton, MA, USA) software.

### 4.3. Laser Irradiation Experiments

Femtosecond laser illumination was implemented by a Ti:Sapphire-based chirped-pulse two-stage amplifier-laser system (Hidra from Coherent Inc., Santa Clara, CA, USA). The amplifier system delivers pulses with 50 fs pulse length at 795 nm central wavelength with up to 10 Hz repetition rate and up to 25 mJ pulse energy. In these laser systems the first stage is regenerative, whereas the second stage is a multipass amplifier. The irradiation in the present experiments was achieved by a single pulse on a certain position of the samples during the experiments by electronical gating of the pump laser of the amplifier stages. In the beam path, the pulse energy was reduced to 5 mJ by using polarization optics (polarizing and half-wave plates) to avoid self-focusing during the propagation, which can damage the laser optics. The beam was focused by a lens with a 50 cm focal length, and the sample was shifted laterally after each illumination with a single pulse. As a result of the laser shot, a crater was formed at the focal spot since the energy density was above the ablation threshold of the material. Raman measurements were always performed directly at the edges of the craters, where the material was intact but exposed. The ablation threshold of the polymer material was investigated in another experiment and was found to be at 0.02 mJ pulse energy, which equals a fluence of 2.83 Jcm^−2^ (these results are to be published elsewhere).

Laser irradiation experiments were performed under vacuum conditions to avoid nonlinear processes in air, such as the self-focusing of the beam and the ionization of the air near the focus. The pressure in the vacuum chamber was in the range of 10^−6^ mbar. Although exposing the polymer to a vacuum can cause chemical changes (e.g., due to evaporation and water loss), it is not assumed to influence the monitored chemical quantities and DC.

### 4.4. Finite Element Simulations

The plasmonic resonance of the 25 × 75 and 25 × 85 gold nanorods embedded into UDMA was modeled by finite element computations (COMSOL Multiphysics, Stockholm, Sweden). The dielectric properties of gold were implemented based on the literature, while the UDMA index of refraction was determined via ellipsometry [22]. The optical cross-sections of the nanorods were extracted by total- and scattered-field methods, and the absorption cross-section was determined based on Joule heating. The wavelength dependence of the absorption cross-section proved plasmonic resonance at 770 nm and 830 nm when the 25 × 75 and 25 × 85 nanorods were illuminated by linearly polarized light with **E**-field oscillation direction along the long axis. Both commercially available nanorods can be proposed to induce plasmon-enhanced phenomena when considering the 795 nm central wavelength of the laser pulse. For this reason, nanorods from both sizes were used to create nanocomposites.

## 5. Conclusions

The effects of intense, single-shot femtosecond laser irradiation, the size and concentration of gold nanorod doping, and the (control) thermal treatment on the degree of conversion of a photopolymerized UDMA-TEGDMA- (3:1) based nanocomposite were studied. It was shown that the presence of the DDT-capped nanorods could enhance the *DC* by 6–15% even when the plasmon resonance of nanorods is not directly excited during the initial photopolymerization process. The degree of conversion that was reached with nanorod doping could either match (with 25 × 75 nanorods) or even surpass (with 25 × 85 nanorods) the *DC* of the thermally-induced polymerization for 30 min at 180 °C. It was also proven that femtosecond laser irradiation (with the investigated conditions, namely a fluence of 2.83 Jcm^−2^ in the investigated area) increased the *DC* by 2–7%, regardless of nanorod doping or antecedent thermal treatment. The best *DC* values (an increase of 12–15%) were always obtained by applying laser irradiation on the polymers that were doped with nanorods, compared to either of the control groups. The low concentration of the nanoparticles and the short duration of the laser pulses (50 fs) that were independently applied after the initial photopolymerization ensured that the effect of the nanoparticles on the conversion was not from thermoplasmonic effects. The results prove that the excited plasmon resonance on the nanorods and high electromagnetic fields can facilitate the breakage of methacrylate C=C double bonds and the formation of crosslinks independently from the primary photopolymerization process.

## Figures and Tables

**Table 1:** Assignment of the sample IDs with the various conditions used in sample preparation and irradiation experiments.

ID	Heat Treatment	Laser Irradiation	Gold Nanorod Size, nm	Gold Nanorod Concentration, mL^−1^
#1	-	-	-	-
#2	-	-	25 × 75	1.9 × 10^12^
#3	-	+	-	-
#4	-	+	25 × 75	1.9 × 10^12^
#5	+	-	-	-
#6	+	-	25 × 75	1.9 × 10^12^
#7	+	+	-	-
#8	+	+	25 × 75	1.9 × 10^12^
#9	-	-	25 × 85	9.5 × 10^11^
#10	-	+	25 × 85	9.5 × 10^11^
#11	-	-	25 × 85	1.9 × 10^12^
#12	-	+	25 × 85	1.9 × 10^12^

**Table 2:** Comparison of the peak parameters (Raman intensity, full width at half maximum (FWHM), and analytical area) for the two characteristic peaks located at 1447 cm^−1^ and 1640 cm^−1^. The values are the average of three spectra measured in three different spots (standard deviation below 3%, as can be seen in Figure 6). The intensity ratio and analytical area ratio were calculated based on these parameters. The DC was calculated based on Equation (4). For sample ID assignments, see Table 1.

ID	Peak at 1447 cm^−1^			Peak at 1640 cm^−1^			1640/1447		DC [%]
	Intensity [a.u.]	FWHM [cm^−1^]	Area [a.u.]	Intensity [a.u.]	FWHM [cm^−1^]	Area [a.u.]	Intensity Ratio [-]	Area Ratio [-]	
Ref.	0.27	22.4	2.03	1.11	11.15	21.52	4.13	3.36	-
#1	0.98	24.41	25.35	0.88	11.63	12.53	0.89	0.49	78.45
#2	0.96	24.26	24.71	0.59	11.83	8.37	0.62	0.34	84.99
#3	0.97	24.5	25.52	0.58	11.85	8.66	0.59	0.34	85.71
#4	0.97	24.6	25.36	0.38	11.95	5.68	0.4	0.22	90.31
#5	0.95	23.35	23.65	0.57	11.56	8.08	0.6	0.34	85.47
#6	0.94	22.92	22.91	0.58	11.98	8.11	0.62	0.35	84.99
#7	0.93	23.2	23.07	0.41	11.9	6.03	0.44	0.26	89.35
#8	0.96	23.52	24.21	0.44	11.96	6.76	0.46	0.28	88.86
#9	0.96	23.92	24.4	0.43	11.34	6.04	0.45	0.25	89.10
#10	0.96	23.97	24.39	0.36	11.89	5.35	0.38	0.22	90.80
#11	0.94	22.81	22.89	0.41	11.63	5.5	0.43	0.24	89.59
#12	0.95	23.93	24.13	0.24	11.6	3.4	0.26	0.14	93.70

## Data Availability

The data presented in this study are available on request from the corresponding author.

## Supplementary Materials

The supporting information can be downloaded at: https://www.mdpi.com/article/10.3390/ijms232113575/s1.
