# Supplementary material for: The Effect of Femtosecond Laser Irradiation and Plasmon Field on the Degree of Conversion of a UDMA-TEGDMA Copolymer Nanocomposite Doped with Gold Nanorods
Source: Int J Mol Sci. 2022 Nov 5;23(21):13575. doi: 10.3390/ijms232113575 (PMC9657072; doi:10.3390/ijms232113575)
Supplement: Supplementary file 1 [file ijms-23-13575-s001.zip › ijms-1975120-supplementary.pdf]

Supplementary Information

The Effect of Femtosecond Laser Irradiation and Plasmon Field on the Degree of Conversion of a UDMA-TEGDMA Copolymer Nanocomposite Doped with Gold Nanorods

Attila Bonyár [1,*], Melinda Szalóki [2], Alexandra Borók [1,3], István Rigó [3], Judit Kámán [3], Shereen Zangana [1], Miklós Veres [3], Péter Rácz [3], Márk Aladi [3], Miklós Ákos Kedves [3], Ágnes Szokol [3], Péter Petrik [4], Zsolt Fogarassy [4], Kolos Molnár [5], Mária Csete [6], András Szenes [6], Emese Tóth [6], Dávid Vas [6], István Papp [3], Gábor Galbács [7], László P. Csernai [3,8,9], Tamás S. Biró [3], Norbert Kroó [3,10] and NAPLIFE Collaboration [3]

1 Department of Electronics Technology, Faculty of Electrical Engineering and Informatics, Budapest University for Economics and Informatics, 1111 Budapest, Hungary
2 Department of Biomaterials and Prosthetic Dentistry, Faculty of Dentistry, University of Debrecen, 4032 Debrecen, Hungary
3 Wigner Research Centre for Physics, 1525 Budapest, Hungary
4 Centre for Energy Research, Institute of Technical Physics and Materials Science (MFA), 1121 Budapest, Hungary
5 Department of Polymer Engineering, Faculty of Mechanical Engineering, Budapest University for Economics and Informatics, 1111 Budapest, Hungary
6 Department of Optics and Quantum Electronics, University of Szeged, 6720 Szeged, Hungary
7 Department of Inorganic and Analytical Chemistry, University of Szeged, 6720 Szeged, Hungary
8 Department of Physics and Technology, University of Bergen, 5007 Bergen, Norway
9 Frankfurt Institute for Advanced Studies, 60438 Frankfurt/Main, Germany
10 Hungarian Academy of Sciences, 1051 Budapest, Hungary
* Correspondence: bonyar.attila@vik.bme.hu; Tel.: +36-(1)-4632758

S1. Heat treatment and DSC control

Reference samples were created where the conversion of the UDMA-TEGDMA copolymer was completed by applying a heat treatment of 30 min at 180ºC. The resulting DSC curve can be seen in Figure S1. Compared to Figure 5b, no exotherm peak can be observed at 158.7 ºC that would mark the breaking of C=C double bonds.

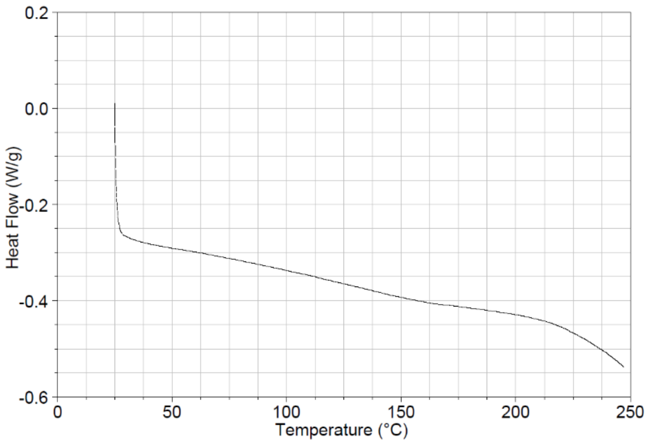

Figure S1. A DSC curve of a UDMA-TEGDMA photopolymer after control heat treatment (180°C for 30 min)

S2. Raman spectra and Lorentzian curve fitting

In the following figures all spectra corresponding tot the 13 different samples are presented, along with the Lorentzian curve fittings on the two evaluated peaks (1447 cm^{-1} and 1639 cm^{-1}). The presented spectra are an average of 3 in every case. Table S1 shows the different experimental conditions for the different samples.

Table S1. Assignment of the sample IDs with the various conditions used in sample preparation and irradiation experiments.

ID	Heat treatment	Laser irradiation	Gold nanorod size, nm	Gold nanorod concentration, mL^{-1}
#0	Pre-polymer mixture (reference sample)			
#1	-	-	-	-
#2	-	-	25 × 75	1.9×10^{12}
#3	-	+	-	-
#4	-	+	25 × 75	1.9×10^{12}
#5	+	-	-	-
#6	+	-	25 × 75	1.9×10^{12}
#7	+	+	-	-
#8	+	+	25 × 75	1.9×10^{12}
#9	-	-	25 × 85	9.5×10^{11}
#10	-	+	25 × 85	9.5×10^{11}
#11	-	-	25 × 85	1.9×10^{12}
#12	-	+	25 × 85	1.9×10^{12}

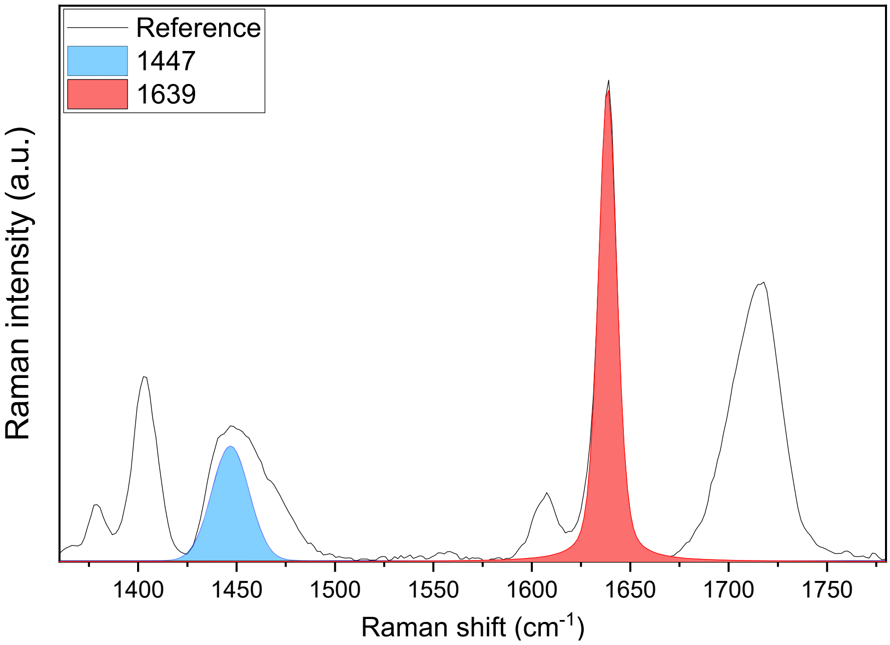

Figure S2. Sample #0 – reference

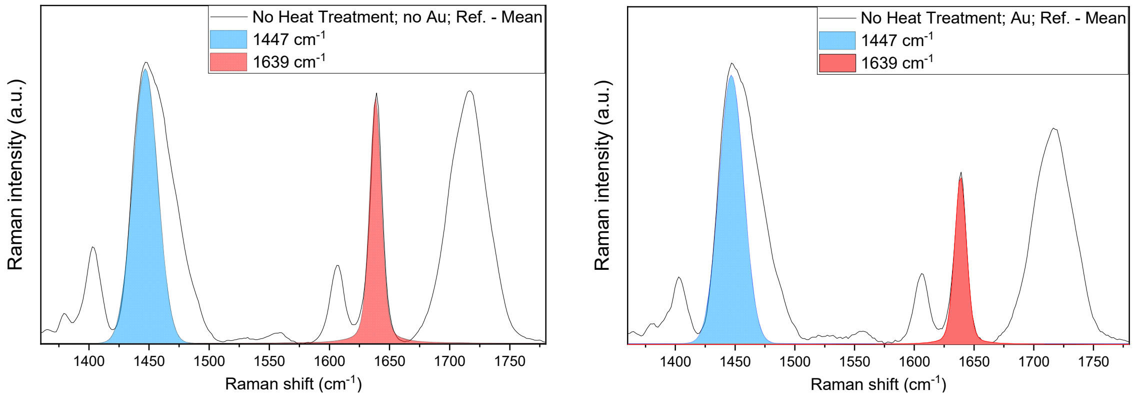

Figure S3. Sample #1 (left) and Sample #2 (right)

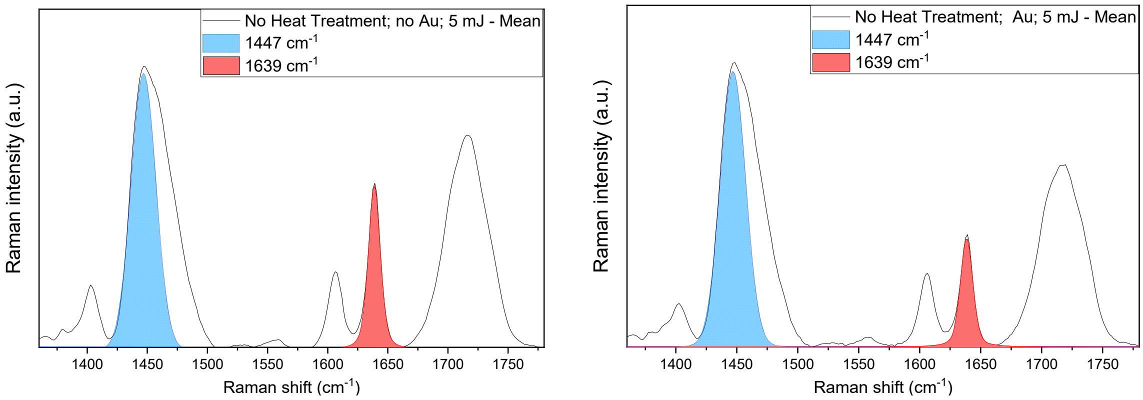

Figure S4. Sample #3 (left) and Sample #4 (right)

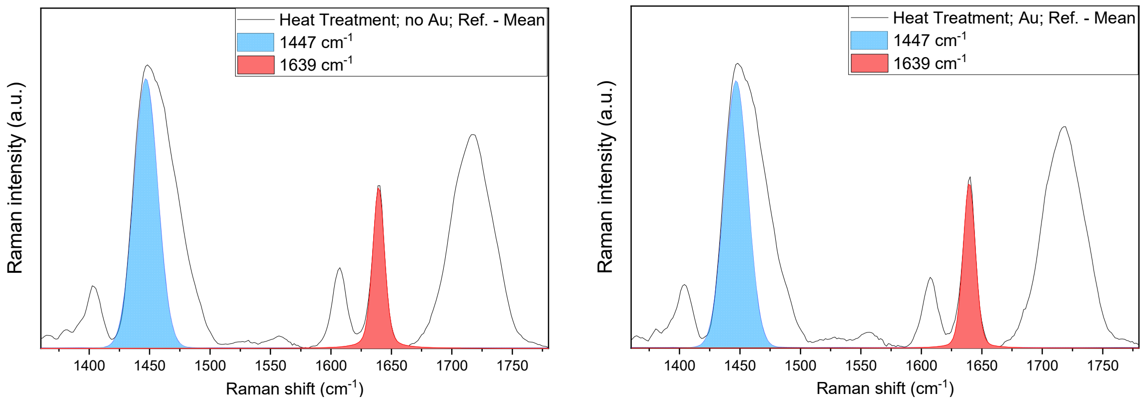

Figure S5. Sample #5 (left) and Sample #6 (right)

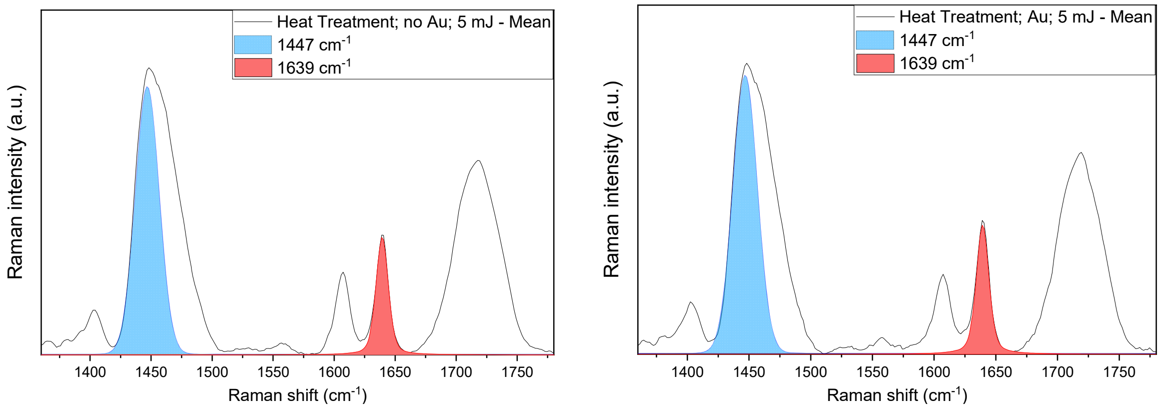

Figure S6. Sample #7 (left) and Sample #8 (right)

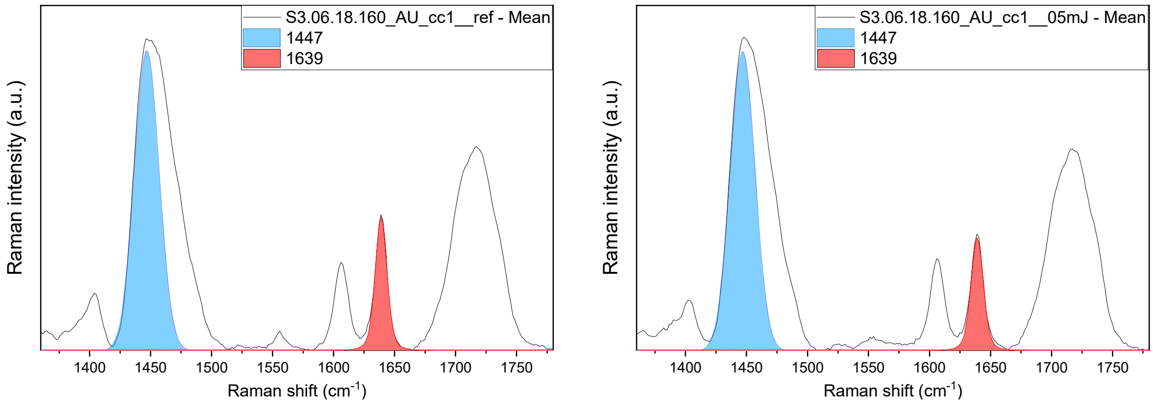

Figure S7. Sample #9 (left) and Sample #10 (right)

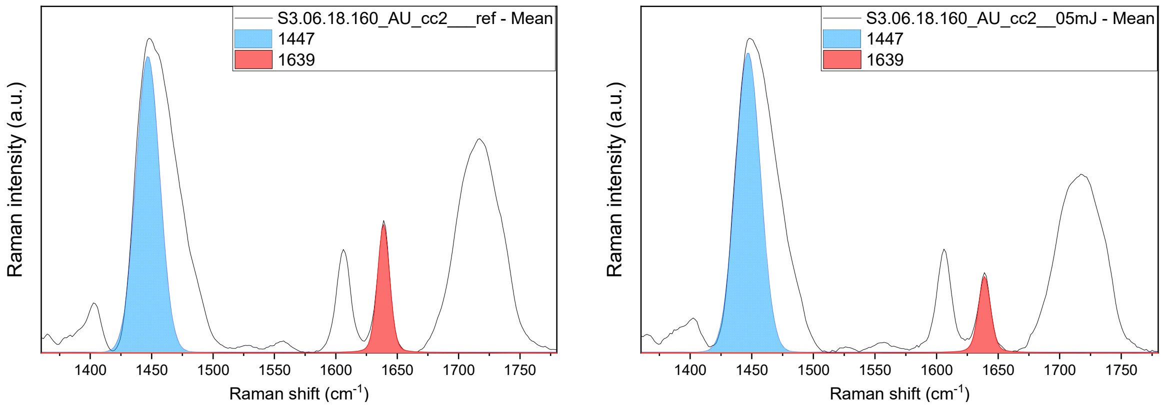

Figure S8. Sample #11 (left) and Sample #12 (right)

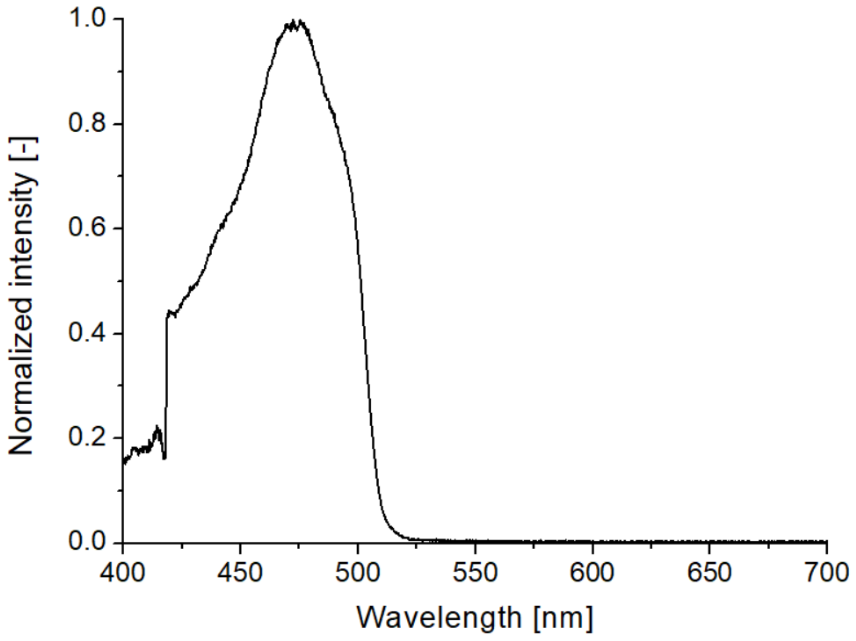

Figure S9. Emission spectrum of the used dental curing lamp.
